# A Case of Perforated Cholecystitis into a Parastomal Hernia

**DOI:** 10.1155/2022/2058051

**Published:** 2022-10-03

**Authors:** Sereibanndith Seang, Amy Hort, Preet K. S. Gosal, Mark Richardson

**Affiliations:** Department of General Surgery, Nepean Hospital, New South Wales, Australia

## Abstract

**Introduction:**

Parastomal hernia is a common complication following an enterostomy. Gallbladder herniation into parastomal hernia is rare and may become symptomatic and inflamed and very rarely can lead to gallbladder perforation. We present the first case of gallbladder perforation inside a parastomal hernia with a unique skin change. *Case Description*. In this report, an 87-year-old female with a history of previous open cystectomy and ileal conduit formation, presented with right upper quadrant pain and worsening parastomal swelling. A computed tomography scan showed a parastomal herniation of the gallbladder, cholelithiasis, and possible early acute cholecystitis. Within 12 hours of admission, bile staining skin changes developed around her urostomy site which raised our suspicion of a perforated gallbladder. Biliary peritonitis was confirmed on laparoscopy the same day. We proceeded with an open midline cholecystectomy without hernia repair. The patient was well at her last follow-up. *Discussion*. A literature review found 14 published cases of incarcerated gallbladder hernia. This uncommon condition mainly affects elderly females. Ten cases were managed operatively, and four cases were managed nonoperatively, with good outcomes. Currently, no consensus on treatment guidelines exists. In a frail elderly patient, a nonoperative approach may be suitable. Operative management was indicated in this case due to clinical concerns of perforation due to developing skin changes. This cutaneous sign has not been previously documented in the literature.

**Conclusion:**

While rare, the gallbladder can herniate and become incarcerated inside a parastomal hernia. Bile staining of the skin should raise clinical suspicion of perforation. Management options depend on patient and pathology factors and can be nonoperative or operative, with or without hernia repair.

## 1. Introduction

A parastomal hernia refers to protrusion of abdominal contents through an abdominal wall defect created during ostomy formation. The reported rates of parastomal hernias in the literature are as high as 65%, with high recurrence rates post repair [1]. Rates are dependent on the type of enterostomy [2]. Usual herniated content includes omentum, small bowel, or colon. Cases of incarcerated parastomal hernias containing the gallbladder are rare, with few reported in the literature [3]. Here, we present the first case of a perforated cholecystitis in a parastomal hernia with a unique clinical sign and outline the workup and management options.

## 2. Case Presentation

An 87-year-old female presented with a 1-day history of right upper quadrant abdominal pain, nausea, and worsening distension of her existing parastomal hernia. She was not constipated, obstipated, or in urinary retention. Her past surgical history of note includes an open cystectomy, total hysterectomy, and formation of ileal conduit for bladder cancer seven years prior. This was followed by a repair of parastomal hernia with prolene onlay mesh three years later. Her parastomal hernia recurred, but she was advised for conservative treatment. Other medical history includes a large head of pancreas lesion and chronic obstructive pulmonary disease.

On examination, her vital signs were within normal limits. A lower midline laparotomy scar was present. Her abdomen was distended with no skin changes, and the right parastomal hernia was incarcerated, measuring 20 by 20 cm, with focal tenderness.

**Figure 1:**
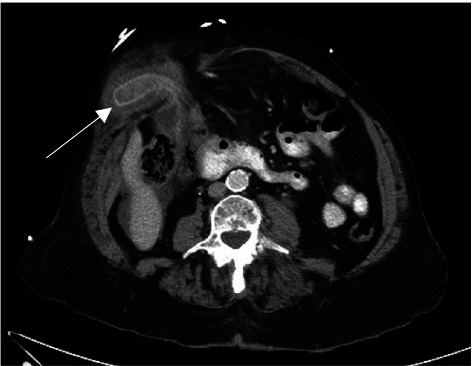
Computed tomography scan of gallbladder fundus herniation into parastomal hernia (arrow) with thickened fundal wall and pericholecystic fluid in axial plane.

**Figure 2:**
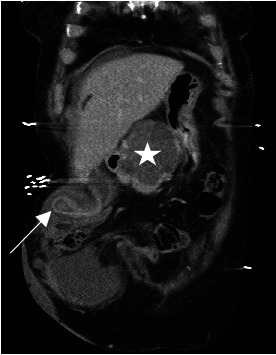
Computed tomography scan of gallbladder herniation (arrow) and large complex cystic head of pancreas lesion (star) in coronal plane.

Her haemoglobin was 111 g/L, white cell count 14.5 × 10^9^/L, C-reactive protein 14 mg/L, lipase 1349 U/L, bilirubin 9 umol/L, and deranged liver enzymes. Computed tomography (CT) demonstrated a parastomal hernia containing a portion of nonobstructed transverse colon and the gallbladder with a thickened fundus, pericholecystic fluid, and impacted stone (Figure 1). The known pancreatic head lesion was stable in size (Figure 2). Given her clinical status and comorbidities, she was kept nil by mouth and a trial of nonoperative management was commenced.

**Figure 3:**
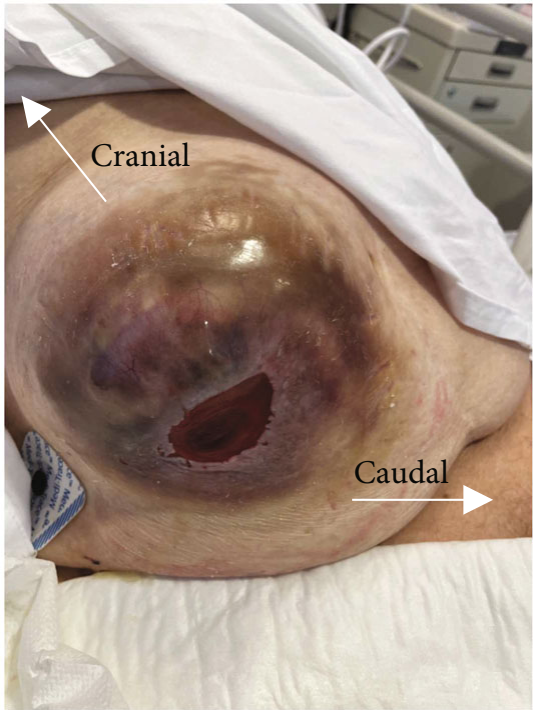
Right-sided parastomal hernia of urostomy site with surrounding bile staining skin which developed within 12 hours of presentation raising concern of gallbladder perforation.

On review that afternoon, her pain worsened and new bile staining surrounding the urostomy site was noted (Figure 3). This raised clinical concerns of perforated acute cholecystitis.

**Figure 4:**
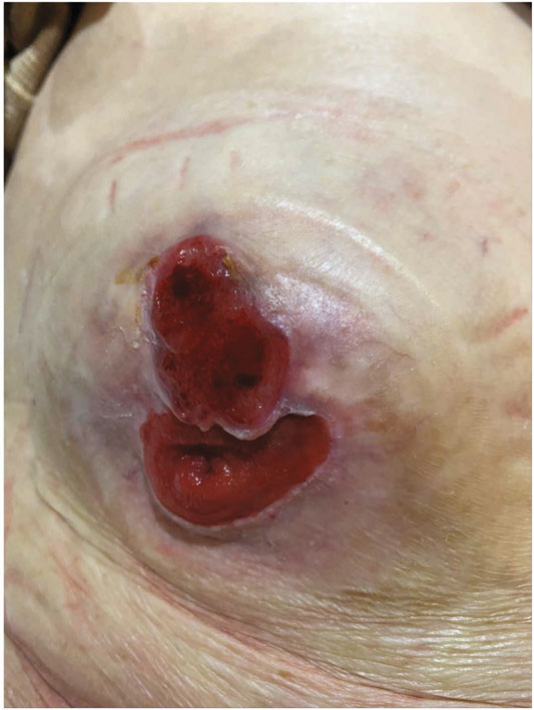
Right-sided parastomal hernia 2-month after discharge. Bile staining skin changes completely resolved. Superior peristomal skin ulceration.

The patient, therefore, was taken for emergency operative intervention without further imaging. This commenced with a diagnostic laparoscopy via a left lower quadrant cut down. This demonstrated extensive adhesions and bilious fluid in the right upper quadrant, making proceeding with laparoscopic management unsafe. An upper midline laparotomy was performed superior to the previous midline scar. An oedematous, perforated gallbladder with biliary peritonitis and spilled yellow gallstones was encountered. The gallbladder was reduced from the hernia, and full-thickness perforation of the fundus was noted. The transverse colon appeared viable, and it was not reduced. Previous mesh was not appreciated. Cholecystectomy was performed, and the parastomal hernia sac was lavaged. A 19 French Blake drain was placed in the right upper quadrant. The operative time was 80 minutes. Postoperatively, she was transferred to the Intensive Care Unit for ongoing care. Her postoperative course was complicated by myocardial infarction, after which she recovered uneventfully. She was discharged 1 month later after a period of inpatient rehabilitation. The skin changes secondary to bile staining completely resolved (Figure 4). Histopathology noted subacute cholecystitis with perforation.

## 3. Discussion

Gallbladder herniation into a parastomal hernia is rare. The majority of cases occur in elderly female patients [3]. Potential contributing factors to gallbladder herniation include loss of visceral fat and elastic tissue, shrinkage of the liver, and lengthening of gallbladder mesentery [4]. The common symptoms are abdominal pain and a tender irreducible parastomal mass. Only one case of gallbladder incarceration in the literature reported overlying skin erythema but not bilious staining [5], while another case with gallbladder perforation did not observe skin changes [6]. Our patient developed bile staining of the skin surrounding her urostomy site, which raised, at this time, our suspicion of gallbladder perforation. To our knowledge, this is the first reported case of this rare entity.

In all but two published cases [7, 8], CT imaging facilitated the diagnosis of gallbladder herniation [3]. Besides feature of cholecystitis, CT can also diagnose perforation, presence of mural or luminal gas, and intestinal obstruction. The approach to management for an incarcerated gallbladder in parastomal hernia is tailored based on patient and pathology factors, as there is no consensus in the literature. High-risk surgical patients may be managed nonoperatively with bowel rest, nasogastric decompression, and intravenous antibiotics, with two cases reporting successful reduction [9, 10]. However, for patients with minimal comorbidities, significant symptoms, or, as demonstrated in this case, cholecystitis with perforation, operative intervention is indicated. This entails open cholecystectomy due to previous abdominal operations with or without hernia repair. One option is to open via a parastomal incision which could facilitate reduction and primary suture repair of the hernia. In this case, an upper midline incision was utilised to avoid her previous laparotomy scar and facilitate safe entry into the abdomen. Our main aim was to perform the cholecystectomy for rapid source control. The hernia defect was not repaired as this would have unnecessarily prolonged operative time in an acutely unstable patient with multiple comorbidities.

One case decompressed the gallbladder intraoperatively with a 14-gauge needle for source control of the intraluminal infection and to facilitate with reduction of the hernia [5]. For noninflamed gallbladder herniation, the hernia may be reduced and repaired without cholecystectomy [11, 12].

The decision to repair the hernia defect is challenging given the high recurrence rate. In an emergency situation, direct fascial repair is simpler but has associated high recurrence rates. Stoma relocation has relatively lower recurrences; however, this prolongs surgery time and risks putting both sites at risk of future hernias and adds potential complications to the ileal conduit. The gold standard repair is with mesh due to its lower recurrences and favourable safety profile [13]. In our case, however, the presence of bilious contamination precluded its use. Our patient could be considered for elective repair or relocation of her parastomal hernia in the future; however, the authors note this had previously been discussed and the perioperative risk outweighed the benefits of a repair.

Here, we present a unique case where a gallbladder-containing parastomal hernia progressed to cholecystitis and perforation. Of note, a rare finding of extensive bile staining of the skin was observed and guided the need for urgent operative intervention. Management options depend on multiple factors and can be nonoperative or operative, with or without repair of the hernia. In the present case, evident perforation of the gallbladder within the hernia prompted surgical exploration.

## Data Availability

Other clinical data or figures supporting diagnosis or management are available from the authors upon request.
